# Artemether-Loaded Zein Nanoparticles: An Innovative Intravenous Dosage Form for the Management of Severe Malaria

**DOI:** 10.3390/ijms22031141

**Published:** 2021-01-24

**Authors:** Yaa Boateng-Marfo, Yuancai Dong, Wai Kiong Ng, Hai-Shu Lin

**Affiliations:** 1 Institute of Chemical and Engineering Sciences, Agency for Science, Technology and Research, 1 Pesek Road, Jurong Island, Singapore 627833, Singapore; nayabdear@gmail.com (Y.B.-M.); dongyuancai@gmail.com (Y.D.); 2 Department of Pharmacy, National University of Singapore, 18 Science Drive 4, Singapore 117543, Singapore; 3 Department of Pharmaceutical Sciences, Sunyani Technical University, P.O. Box 206 Sunyani, Ghana; 4 College of Pharmacy, Shenzhen Technology University, 3002 Lantian Road, Pingshan District, Shenzhen 518118, China

**Keywords:** artemether, artemisinin, extended release, hemolysis, intravenous, nanoparticles, pharmacokinetics, severe malaria, sodium caseinate, zein

## Abstract

Artemether, an artemisinin derivative, is used in the management of life-threatening severe malaria. This study aimed to develop an intravenous dosage form of artemether using nanotechnology. Artemether-loaded zein nanoparticles were prepared by modified antisolvent precipitation using sodium caseinate as a stabilizer. Subsequently, the physicochemical properties of the nanoparticles were characterized; the in vitro hemolytic property was examined with red blood cells, while the pharmacokinetic profile was evaluated in Sprague–Dawley rats after intravenous administration. The artemether-loaded zein nanoparticles were found to display good encapsulation efficiency, excellent physical stability and offer an in vitro extended-release property. Interestingly, encapsulation of artemether into zein nanoparticles substantially suppressed hemolysis, a common clinical phenomenon occurring after artemisinin-based antimalarial therapy. Upon intravenous administration, artemether-loaded zein nanoparticles extended the mean residence time of artemether by ~80% in comparison to the free artemether formulation (82.9 ± 15.2 versus 45.6 ± 16.4 min, *p* < 0.01), suggesting that the nanoparticles may prolong the therapeutic duration and reduce the dosing frequency in a clinical setting. In conclusion, intravenous delivery of artemether by artemether-loaded zein nanoparticles appears to be a promising therapeutic option for severe malaria.

## 1. Introduction

Malaria is a life-threatening tropical disease caused by *Plasmodium* parasites that are transmitted to people through the bites of infected female Anopheles mosquitoes [1,2,3]. According to World Malaria Report 2020, there were 229 million cases worldwide and more than 400,000 people died of malaria in 2019 [3]. Children under the age of five are the most vulnerable group affected by malaria and they accounted for 67% of global malaria deaths [3]. It is generally believed that malaria is associated with poverty and sometimes it is even the cause of poverty [4,5].

**Figure 1:**
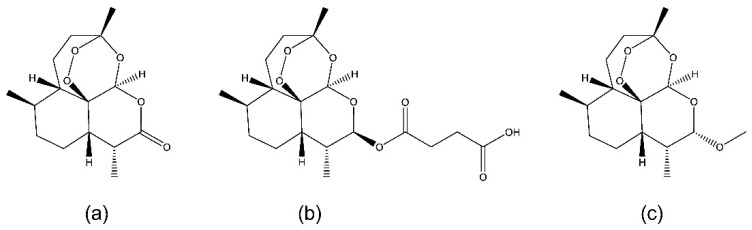
Chemical structures of artemisinin (**a**), artesunate (**b**) and artemether (**c**).

The management of malaria has been revolutionarily improved by the introduction of the artemisinin derivatives in the 1990s, a group of semisynthetic compounds produced from artemisinin (Figure 1a), a sesquiterpene lactone endoperoxide originally isolated from the traditional Chinese herb *Artemisia annua* [1,6]. As artemisinin derivatives are rapidly effective, safe and well tolerated, artemisinin-based combination treatments are recommended by the World Health Organization (WHO) as first-line therapies in all malaria endemic countries [1,7]. The discovery of artemisinin has saved millions of lives and was acknowledged by the award of the Nobel Prize in Physiology or Medicine to Professor Youyou Tu in 2015 [1].

As the mortality from untreated severe malaria can be as high as 100%, severe malaria is a medical emergency, and requires intensive nursing care and careful management [2,7]. Although artemisinin derivatives such as artesunate (Figure 1b) and artemether (Figure 1c) can be given through oral administration to treat uncomplicated malaria [7], intravenous or intramuscular administration of artesunate is the first choice for the management of severe malaria [2,7]. In case injectable artesunate is not available, parenteral artemether is an alternative option [7]. Different from artesunate, artemether displays limited aqueous solubility and its parenteral formulation is only available as a premixed oil-based solution for intramuscular injection [8]. When dosed intramuscularly, artemether may be absorbed slowly but erratically, resulting in a smaller survival benefit than parenteral artesunate [2,7]. Clearly, it of great clinical significance to formulate artemether into an intravenous dosage form for the management of severe malaria.

During the past two decades, advances in nanotechnology have made great contributions to pharmaceutical industries. The formulation development of paclitaxel, a water insoluble chemotherapeutic agent with broad-spectrum activity in many solid tumors is an excellent example [9]. Because of the solubility issue, paclitaxel is formulated into parenteral dosage form with polyethoxylated castor oil (Cremophor^®^ EL) and ethanol in the conventional formulation (Taxol^®^; CrEL-paclitaxel) [9]. However, due to vehicle toxicity, this formulation is commonly associated with adverse effects such as hypersensitivity reactions, neutropenia and neuropathy [9]. Abraxane^®^, the 130 nm human serum albumin-bound paclitaxel (nab-paclitaxel), is the first parenteral nanoparticle formulation approved for clinical application in medical history [9]. Since Abraxane^®^ selectively delivers larger amounts of paclitaxel to tumors while avoiding some of the solvent related toxicities of the conventional formulation, it displays clinical efficacy and safety superior to the conventional formulation [9]. Clearly, protein-based nanoparticles are a practical strategy for parenteral delivery of water insoluble therapeutic agents.

In our recent study, artemether was formulated into an intravenous dosage form using nanoparticle albumin bound technology [10]. Such a formulation not only enhanced the dissolution of artemether, but also decreased hemolysis [10], a common clinical phenomenon occurred after artemisinin-based therapy [11]. However, as malaria is a disease of poverty [4,5], excipients with higher affordability are more appropriate for the intravenous delivery of antimalaria agents. Zein, a plant protein obtained from corn, has attracted significant interest in the biomedical community over the past decade [12,13]. As it is biodegradable, biocompatible and cost-effective, zein appears to be a promising excipient [12,13]. In the present study, artemether-loaded zein nanoparticles were prepared by modified antisolvent precipitation using sodium caseinate as a stabilizer. Subsequently, their physicochemical properties were characterized; the in vitro hemolytic property was examined with fresh red blood cells, while the pharmacokinetic profile was evaluated in Sprague–Dawley rats after intravenous administration. Hopefully, the information obtained from this study will facilitate the development of an innovative therapeutic option for severe malaria.

## 2. Results

### 2.1. Preparation of Artemether-Load Zein Nanoparticles

**Figure 2:**
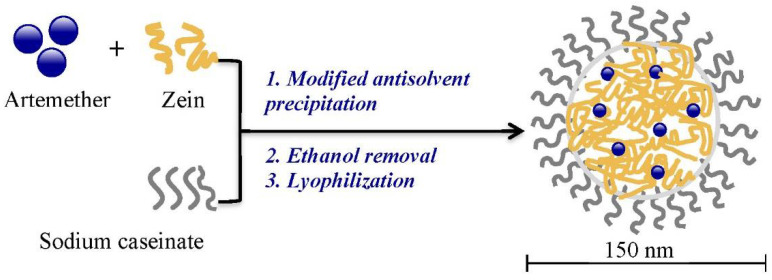
Preparation of artemether-loaded zein nanoparticles.

As displayed in Figure 2, artemether-load zein nanoparticles were prepared by modified antisolvent precipitation using sodium caseinate as a stabilizer. When aqueous sodium caseinate solution was added to the ethanol solution with artemether and zein, ethanol content reduced sharply, providing a constant microenvironment for the formation of nanoparticles with more ordered structural arrangements of component molecules. It also led to the production of nanoparticles with monomodal distribution and narrow particle size distribution [14]. On the contrary, dropwise addition of ethanol solution to aqueous solution results in particles with a less ordered structural arrangement and multimodal particle size distribution. It also causes increased exposure of the hydrophobic amino acids in zein molecules and therefore requires more stabilizer in order to stabilize the nanoparticles [14].

### 2.2. Optimization of Formulation and Process Parameters

#### 2.2.1. Sodium Caseinate/Zein Mass Ratio

**Figure 3:**
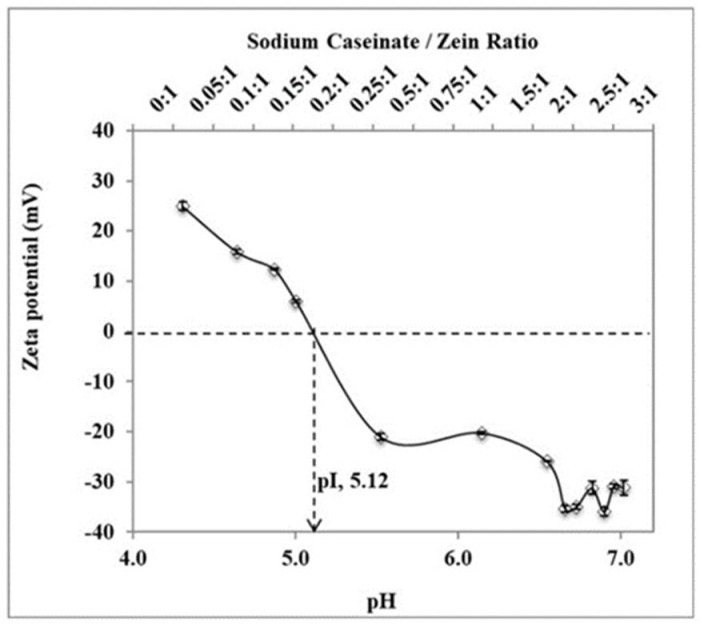
A plot of zeta potential against pH as sodium caseinate/zein ratio varied from 0:1 to 3:1. Zeta potential shifted from positive values to negative values as sodium caseinate/zein ratio increased. Isoelectric point (pI, pH at which zeta potential is zero) was extrapolated to be 5.12. The experimental data were obtained from the nanoparticles prepared from three different batches (*n* = 3); symbols represent mean values while error bars represent standard deviation (SD).

**Figure 4:**
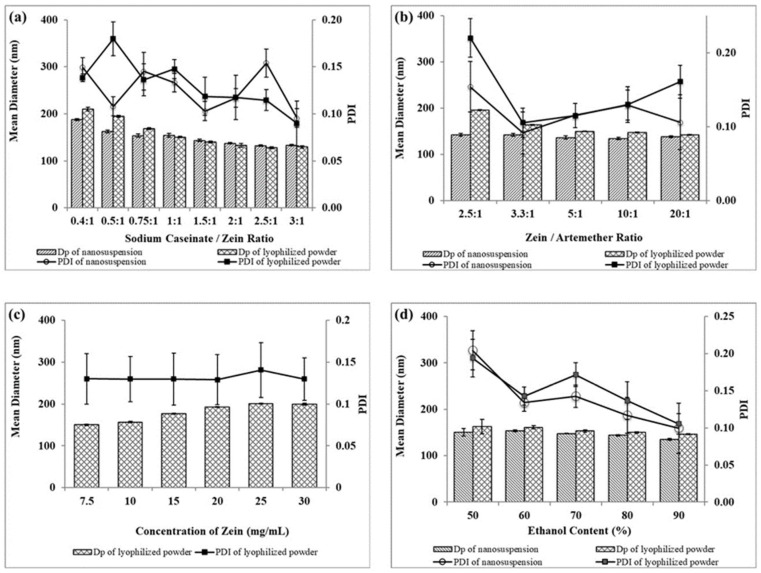
Effects of formulation variables (**a**) sodium caseinate/zein ratio, (**b**) zein/artemether ratio, (**c**) concentration of zein and (**d**) ethanol content on particle size and polydispersity index (PDI) of artemether-loaded zein nanoparticles in nanosuspension and lyophilized powder forms. The experimental data were obtained from the nanoparticles prepared from three different batches (*n* = 3); symbols represent mean values while error bars represent SD.

Sodium caseinate serves as a stabilizer and cryoprotectant in this work. It also enhances the cellular uptake of nanoparticles [15]. Sodium caseinate is amphiphilic and charged in nature. It adsorbs to the surface of nanoparticles and stabilizes them by both electrostatic repulsion and steric hindrance—i.e., electrosteric stabilization [16,17]. As shown in Figure 3, zeta potential shifted from positive values to negative values as the sodium caseinate/zein concentration increased. This is evidence of stabilization by electrostatic repulsion between nanoparticles. Moreover, a further decrease in particle size occurred by increasing the sodium caseinate/zein ratio above 0.75:1 (Figure 4a). Since the zeta potential was higher than −30 mV at a sodium caseinate/zein ratio of 0.75:1, the decrease in particle size with a higher sodium caseinate/zein ratio is suggestive of stabilization by steric hindrance.

**Figure 5:**
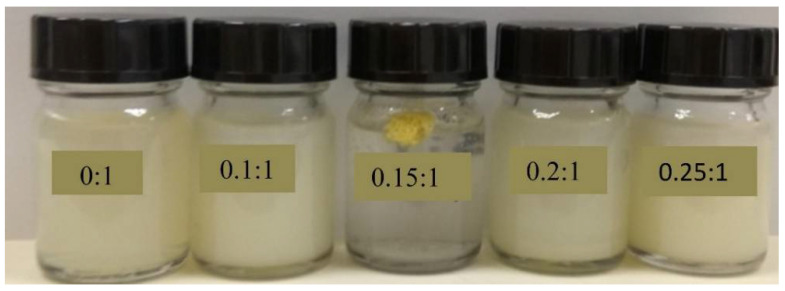
Physical appearances of artemether-loaded zein nanosuspension (before ethanol removal) prepared using sodium caseinate/zein ratio from 0:1 to 0.25:1.

The effects of the sodium caseinate/zein ratio on the size of artemether-loaded zein nanoparticles were studied by varying the sodium caseinate/zein ratio from 0:1 to 3:1. The physical appearance of artemether-loaded zein nanosuspension (before ethanol removal) prepared using a sodium caseinate/zein ratio from 0:1 to 0.25:1 is shown in Figure 5. At a caseinate/zein ratio of 0:1 to 0.1:1, relatively larger particles were formed. When the sodium caseinate/zein ratio was increased to 0.15:1, an extensive coagulation of zein occurred forming a big yellow lump in a clear medium. This is due to the high positive charge of zein, which dominated the system at a sodium caseinate/zein ratio of 0:1 to 0.1:1, with zeta potential values of +25 to +16 mV. As the sodium caseinate/zein ratio increased to 0.15:1, there was an extensive neutralization of the positive charge of zein giving a zeta potential value of +6 mV and pH of 5. From a plot of zeta potential against pH (Figure 3), the *x*-intercept was extrapolated to be 5.12, representative of isoelectric point (pI) of the system. Zein has a high tendency to aggregate in water because its original pI of 6.2 is closer to neutral pH [18]. The presence of sodium caseinate (pI: 4.5) [19] shifted the pI of the artemether-loaded zein nanoparticles further away from neutral pH to 5.12, reducing the tendency of the nanoparticles to aggregate in aqueous environments. The lack of charge on the artemether-loaded zein nanoparticles at the pI is responsible for the extensive coagulation of zein, forming a lump a clear medium. Further increase in sodium caseinate/zein ratio above 0.15:1 led to a shift in zeta potential to negative values, which conferred stability on the nanoparticles by minimizing aggregation. Though artemether-loaded zein nanoparticles prepared at a sodium caseinate/zein ratio of 0.25:1 were stable with a zeta potential value of −20 mV, nanoparticles prepared at sodium caseinate/zein ratio below 0.4:1 were not redispersible after lyophilization. This indicated that a higher caseinate/zein proportion was required for cryoprotection during lyophilization.

A sharp decrease in particle size from 210 ± 4 to 150 ± 2 nm was observed as the sodium caseinate/zein ratio increased from 0.4:1 to 1:1. This was followed by a gradual decrease to 129 ± 2 nm as the sodium caseinate/zein ratio increased to 3:1, as shown in Figure 4a. Clearly, a sodium caseinate/zein ratio ≥0.4:1 was sufficient for cryoprotection. Therefore, there was no significant difference between the particle size of the nanosuspensions and that of the lyophilized powders. Particle size decreased with an increasing sodium caseinate/zein ratio because higher sodium caseinate proportions increased their stabilizing effect on the nanoparticles. Varying the sodium caseinate/zein ratio from 0.4:1 to 3:1 did not significantly affect the polydispersity index (PDI) in any order. PDI values between 0.10 and 0.18, as presented in Figure 4a, are indicative of particles with a narrow size distribution. Sodium caseinate/zein ratio 1:1 was selected for the subsequent optimization steps.

#### 2.2.2. Zein/Artemether Mass Ratio

The impacts of the zein/artemether ratio on size and PDI of artemether-loaded zein nanoparticles are presented in Figure 4b. When the zein/artemether ratio increased, the proportion of the drug decreased, resulting in smaller particles. This was evident in the decrease in particle size from 196 ± 1 to 142 ± 3 nm as the zein/artemether ratio was increased from of 2.5:1 to 20:1. Nanoparticles prepared without artemether exhibited a size of 139 ± 1 nm. This was probably due to the fact that as the zein/artemether ratio increased, the same amount of zein incorporated in the decreasing amount of artemether. In addition, decreasing the proportion of artemether increased the stabilizing effect of sodium caseinate on nanoparticles resulting in less aggregation. The PDI of nanoparticles fell between 0.09 ± 0.03 and 0.22 ± 0.03 and did not follow any particular order. A zein/artemether ratio of 5:1 was used for the subsequent optimization steps.

#### 2.2.3. Zein Concentration

The impact from zein concentration on particle size was examined by varying it from 7.5 to 30 mg/mL, while keeping the zein/artemether ratio and sodium caseinate constant. As shown in Figure 4c), when the zein concentration increased from 7.5 to 25 mg/mL, particle size increased from 150 ± 2 to 201 ± 1 nm. Further increase in zein concentration to 30 mg/mL did not result in any change in particle size. Increasing zein concentration resulted in more zein and artemether molecules coming together to form larger particles. This is because in order to accommodate the increasing amount of zein and artemether in the colloidal system, larger particles were formed to reduce the overall area to volume ratio of the nanosuspension [20]. Increasing zein concentration also led to an increase in the viscosity of the nanosuspension, resulting in the formation of larger particles during nucleation. Similar observations have been reported before [20,21]. The PDI was observed to be below 0.14 ± 0.03.

#### 2.2.4. Ethanol Concentration

The impacts of ethanol concentration on the size and PDI of artemether-loaded zein nanoparticles are presented in Figure 4d. Increasing the ethanol content from 50 to 90% *v*/*v* led to a decrease in particle size of the nanoparticles from about 163 ± 16 to 140 ± 1 nm. PDI also showed a similar pattern with the smallest particle observed at 90%. The higher the solubility of zein in an ethanol–water binary solvent system, the smaller the particle size of nanoparticles produced [22]. The results indicated that zein was most soluble with a 90% ethanol content. Since there was no significant difference between the size of nanoparticles obtained at 80 and 90% ethanol contents, the former was selected for next optimization steps.

In summary, higher sodium caseinate/zein mass ratio, higher zein/artemether mass ratio, lower zein concentration and higher ethanol content favored the formation of smaller nanoparticles.

### 2.3. Drug Content and Encapsulation Efficiency

To evaluate the drug loading capacity and encapsulation efficiency, the artemether-loaded zein nanoparticles were prepared using a sodium caseinate/zein mass ratio of 1:1, a zein concentration of 7.5 mg/mL, an ethanol concentration of 80% (*v*/*v*) and zein/artemether ratios varying from 2.5:1 to 20:1. The results are listed in Table 1.

### 2.4. Physical Characterization

The following preparation parameters, i.e., a sodium caseinate/zein mass ratio of 1:1, a zein/artemether mass ratio of 5:1, a zein concentration of 7.5 mg/mL and an ethanol concentration of 80% (*v*/*v*), were chosen to prepare artemether-load zein nanoparticles for further physical characterization using in vitro and in vivo testing.

#### 2.4.1. Particle Morphology

**Figure 6:**
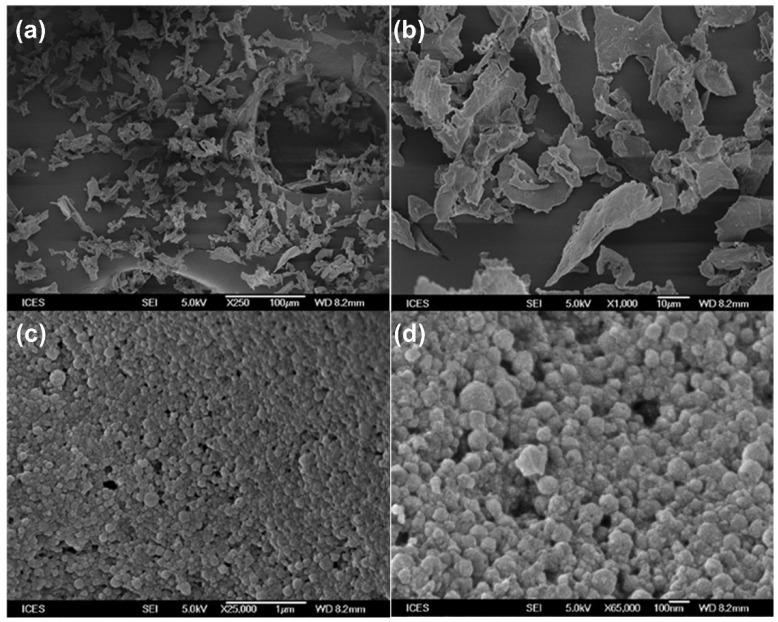
Scanning electron microscopic images of artemether-loaded zein nanoparticles powder at different magnifications. Image (**a**): 250×; (**b**): 1000×; (**c**) 25,000×; (**d**): 65,000×.

The morphology of the typical artemether-load zein nanoparticles powder was examined and is presented in Figure 6. The nanoparticles appeared leafy at lower magnifications (250× and 1000×). At higher magnifications, the nanoparticles were observed to be spherical with rough surfaces. Particle size was estimated to be around 150 nm confirming particle size analyses by dynamic light scattering. Nanoparticles prepared with different formulation parameters reported in this work exhibited similar morphologies (images not shown).

#### 2.4.2. Thermal Analysis

**Figure 7:**
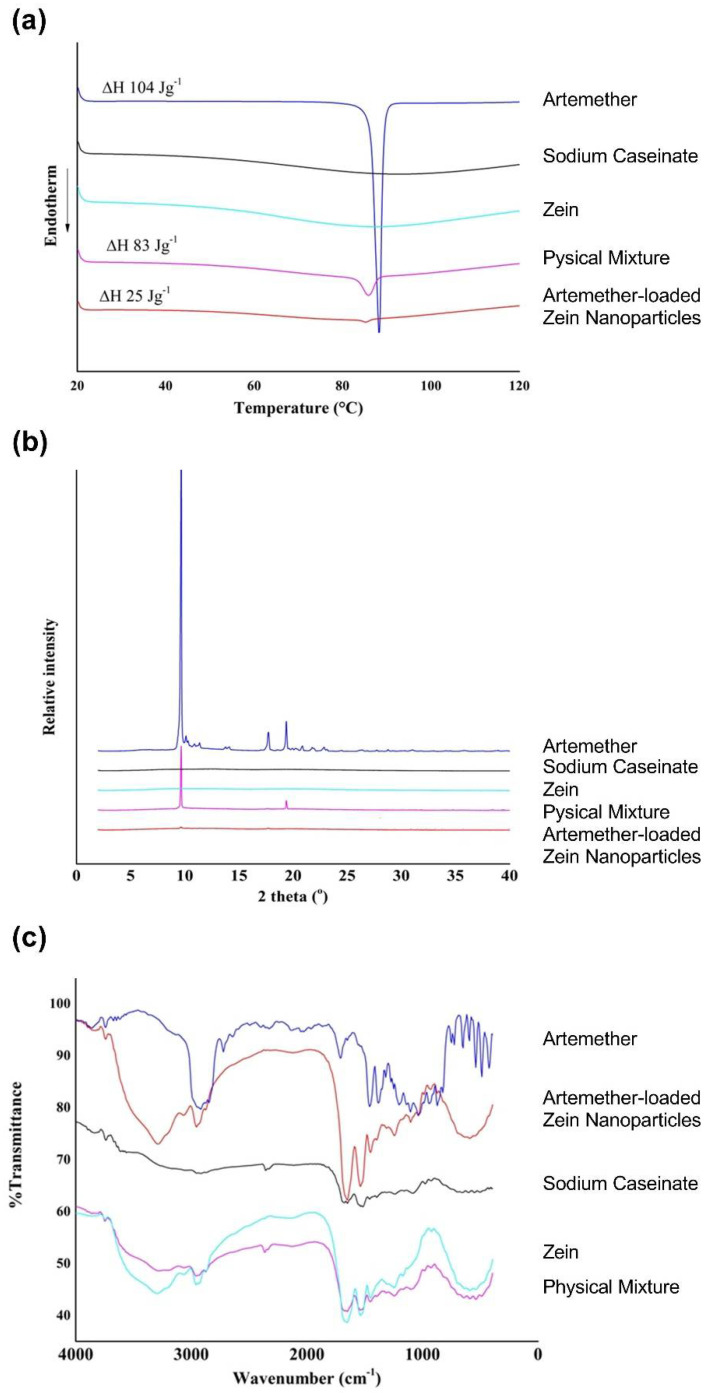
Physical characterization of artemether-loaded zein nanoparticles. Image (**a**) thermograms; (**b**) X-ray powder diffractograms; (**c**) Fourier transform infrared spectra of raw artemether, artemether-loaded zein nanoparticles, sodium caseinate, zein and their physical mixture (mass ratio of 1:5:5).

The thermograms of raw artemether, artemether-loaded zein nanoparticles, sodium caseinate, zein and their physical mixture (mass ratio of 1:5:5) are displayed in Figure 7a. Zein and sodium caseinate did not exhibit any observable thermal peaks, implying that no physical transition occurred in the investigated range. Raw artemether exhibited the characteristic melting peak at 88 °C with an enthalpy of 104 J·g^−1^. In the thermogram of the physical mixture, the endothermic peak of artemether was broad and shifted to 86 °C with an enthalpy of 83 J·g^−1^. This shift and broadening of the endothermic peak together with the slight reduction in enthalpy observed were probably due to the presence of zein and sodium caseinate, which served as impurities. The melting peak in the thermogram of artemether-loaded zein nanoparticles was broadened and shifted to 85 °C. In addition, its enthalpy was decreased to 25 J·g^−1^, indicating significant amorphization of artemether.

#### 2.4.3. Crystallinity

The X-ray powder diffractograms of raw artemether, artemether-loaded zein nanoparticles, sodium caseinate, zein and their physical mixture (mass ratio of 1:5:5) are displayed in Figure 7b. Zein and sodium caseinate did not show any distinct peaks due to their amorphous natures. Raw artemether exhibited two distinct peaks at 2θ values of 9.66° and 19.38°, which are characteristic of crystalline artemether. These peaks were also observed in the physical mixture. No characteristic artemether peak was observed in the artemether-loaded zein nanoparticles, suggesting extensive amorphization of artemether in the nanoparticles. This agrees with the results observed in the thermal analysis.

#### 2.4.4. Fourier Transform Infrared (FTIR) Spectra

The FTIR spectra of raw artemether, artemether-loaded zein nanoparticles, sodium caseinate, zein and their physical mixture (mass ratio of 1:5:5) are shown in Figure 7c. Characteristic artemether bands were recorded at 2937, 1455 and 1036 cm^−1^, representative of C-H stretching, C-H bending and C-O-C stretching vibrations, respectively [23]. The FTIR spectra of artemether-loaded zein nanoparticles showed peaks at 2947 (C-H stretching vibration), 1450 (C-H bending vibration) and 1034 cm^−1^ (C-O-C stretching vibration). The presence of these characteristic artemether bands in the artemether-loaded zein nanoparticles suggests that artemether remained intact in the formulation and underwent insignificant degradation.

### 2.5. Release Profile

**Figure 8:**
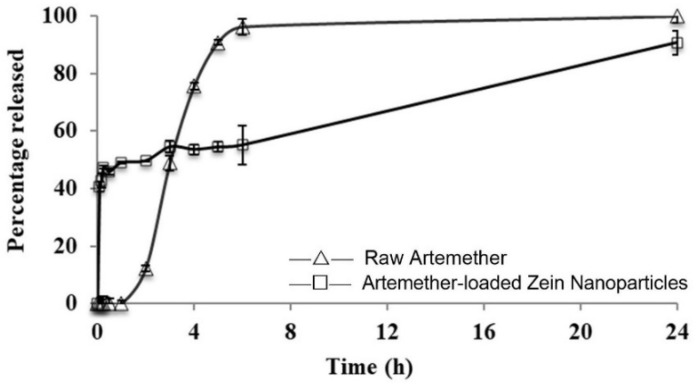
Release profiles of artemether from artemether-loaded zein nanoparticles and raw artemether in phosphate buffer solution (PBS) at 37 °C. All experiments were carried out in triplicate (*n* = 3); symbols represent mean values while error bars represent SD.

The release profile of artemether-loaded zein nanoparticles is presented in Figure 8. Artemether was found to be released from the nanoparticles through a biphasic pattern with an initial burst release phase, followed by a slow-release phase. Artemether of 47 ± 1% was released in the first 15 min. The release was gradually increased to 99 ± 2% in 24 h. The initial rapid release represented the burst release of unencapsulated artemether in the formulation. Encapsulated artemether was released slowly, accounting for the second phase in the release profile since drug molecules were embedded in a matrix with zein in the nanoparticle structure. The extended-release property of the nanoparticles could be attributed to the hydrophobic nature of zein, which slowed down the penetration of water into the nanoparticles. Unlike another study [24], the unencapsulated drug was allowed to remain in the formulation. This is because a high initial burst release is beneficial in terms of achieving a therapeutic concentration of artemether in the bloodstream immediately after administration. Probably due to inferior aqueous solubility, raw artemether exhibited very slow dissolution in the first 2 h. However, almost all raw artemether was dissolved within 5 h while only 54 ± 2% of artemether was released from the nanoparticles during the same period. The biphasic and extended-release behaviour of artemether-loaded zein nanoparticles may lead to better therapeutic efficacy in clinical settings for severe malaria patients under emergency.

### 2.6. Physical Stability

**Figure 9:**
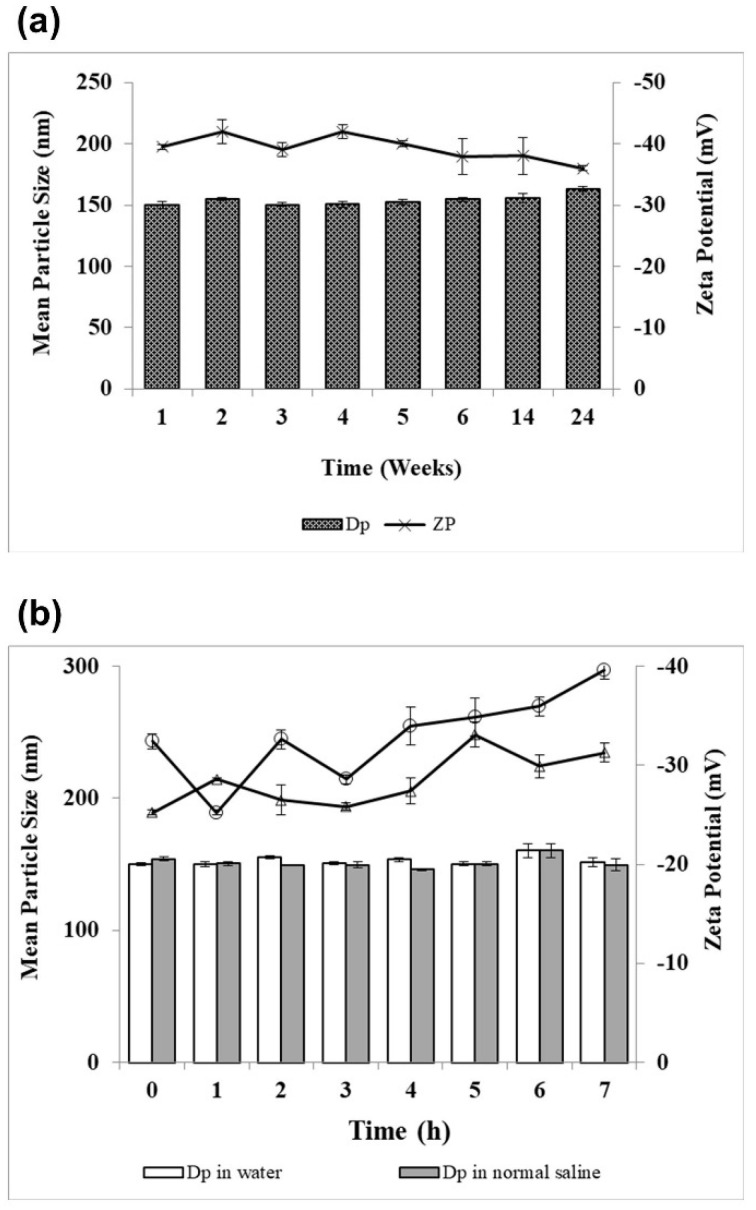
Physical stability of artemether-loaded zein nanoparticles. (**a**) Particle size (DP) and zeta potential (ZP) analyses of artemether-loaded zein nanoparticles powder on storage at 4 °C and relative humidity of 75 ± 5% for 24 weeks; (**b**) particle size and zeta potential analyses of artemether-loaded zein nanoparticles after reconstitution with water and with normal saline. All experiments were carried out in triplicate (*n* = 3); symbols represent mean values while error bars represent SD.

Physical stability of lyophilized artemether-loaded zein nanoparticles powder on storage and after reconstitution with either Milli-Q water or normal saline was examined. The study was performed by assessing change in particles size, zeta potential and encapsulation efficiency. All zeta potential measurements were taken at pH 6.3 and 5.9 for powder reconstituted with Milli-Q water and normal saline, respectively. Electrostatic repulsion between nanoparticles measured as zeta potential stabilizes them by preventing aggregation and particle growth. Stable colloidal systems possess zeta potential values above −25 mV [25]. When artemether-loaded zein nanoparticles were stored at 4 °C and 75% relative humidity for 24 weeks, the encapsulation efficiency remained unchanged; the particle size remained below 160 nm, while the zeta potential fell in the range of −40 ± 2 mV throughout the storage duration (Figure 9a), suggesting their excellent physical stability.

Artemether-loaded zein nanoparticles also showed a high level of stability after reconstitution with Milli-Q water or normal saline as no significant increase in particle size was found (Figure 9b). The size of artemether-loaded zein nanoparticles remained below 160 nm for 7 h after reconstitution with Milli-q water or normal saline. Zeta potential values of artemether-loaded zein nanoparticles reconstituted with normal saline were generally lower than that of Milli-Q water. Normal saline has a high ionic strength (154 mEq/L of Na^+^ and Cl^−^) that exerted shielding effect on nanoparticles after reconstitution, hence weakening the interparticular electrostatic repulsive forces. This could explain for the lower zeta potential values of normal saline reconstituted products [26].

### 2.7. In Vitro Hemolysis Test

As hemolysis is a common clinical phenomenon occurring after artemisinin-based therapy [11], it is of great interest to examine the hemolytic property of the artemether-loaded zein nanoparticles. An in vitro hemolysis test is commonly used to study the toxicity of a formulation on red blood cells (RBCs). It can also be used to estimate potential membrane damage caused by a parenteral dosage form [27]. The breakdown of RBCs is measured by the amount of haemoglobin released. Hemolytic tendencies of artemether-loaded zein nanoparticles (artemether: 2 mg/mL), 5% DMSO, sodium caseinate (7.5 mg/mL), sodium caseinate together with artemether (2 mg/mL) and artemether (2 mg/mL) in 5% DMSO were investigated. Phosphate buffered saline (PBS) and Triton X 100 were used as the negative and positive controls, respectively, as (1) PBS is assumed to cause no haemolysis and (2) Triton X 100 is a surfactant known to cause 100% hemolysis. Since artemether is insoluble in water, artemether was dissolved in 5% DMSO.

**Figure 10:**
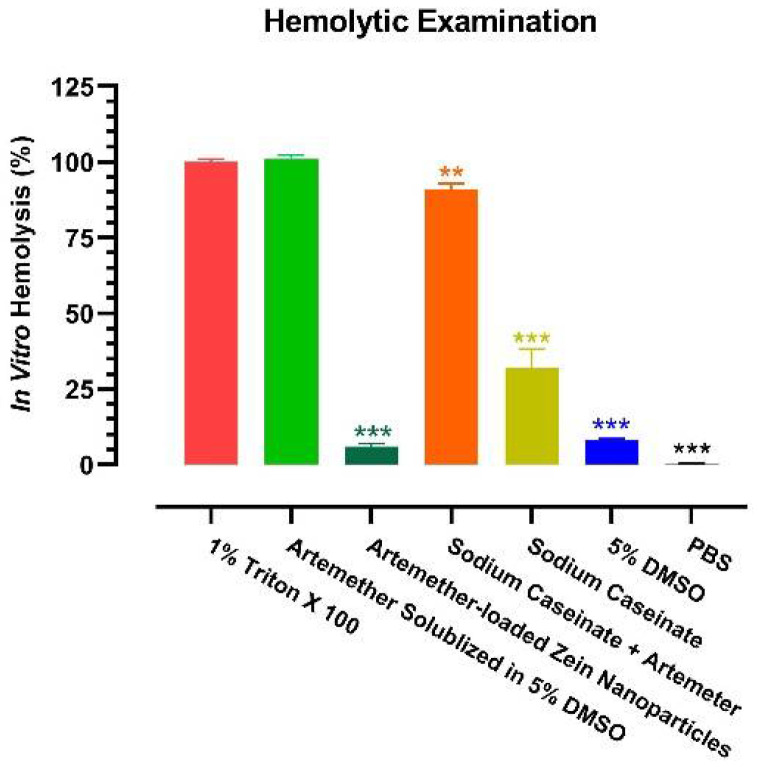
Hemolytic property of artemether-loaded zein nanoparticles. The hemolytic tendency was examined by in vitro hemolysis test. All experiments were carried out in triplicate (*n* = 3); symbols represent mean values while error bars represent SD. ** *p* < 0.01, *** *p* < 0.001 between this group and 1% Triton X 100, two-tailed unpaired *t*-test.

Consistent with the clinical observations [11], artemether cased substantial hemolysis (Figure 10). Although 5% DMSO had a mild hemolytic effect (8.0 ± 0.6%), when artemether was solubilized in 5% DMSO, it destroyed RBCs as much as the positive control. Of note, sodium caseinate also caused significant hemolysis (32.0 ± 6.2%). However, when the hemolytic artemether and sodium caseinate were formulated into nanoparticles with zein, the hemolytic effect dropped by at least 15-fold (6.0 ± 1.0%). Obviously, a less hemolytic intravenous dosage of artemether was enabled by the nanoparticles.

### 2.8. Pharmacokinetic Study

**Figure 11:**
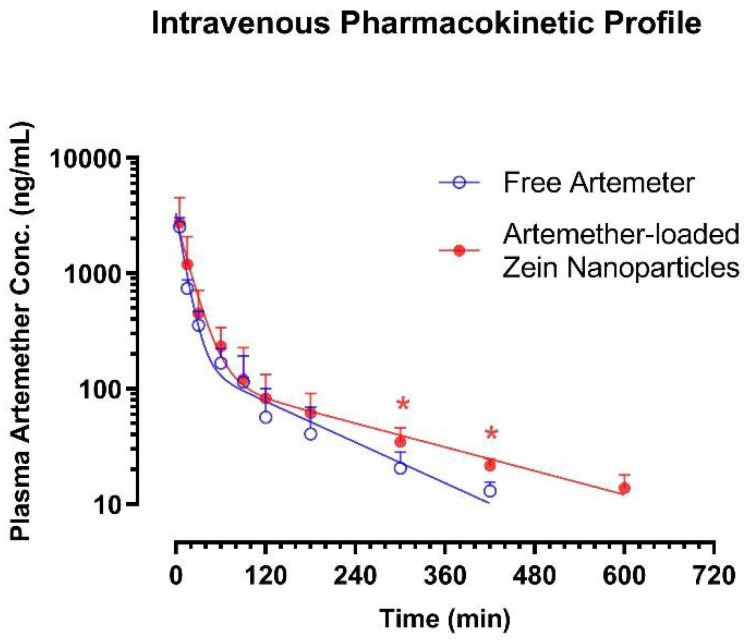
Intravenous pharmacokinetic profiles of artemether. The male Sprague–Dawley rats received respective intravenous administration of artemether in nanoparticles or cosolvent formulation at 5 mg/kg. Data are presented as mean ± SD; *n* = 5 except *n* = 3 at 420 min for rats that received artemether in cosolvent. * *p* < 0.05 between nanoparticle group and cosolvent group, two-tailed unpaired *t*-test.

The pharmacokinetic profiles of artemether were assessed after single bolus intravenous administration at the dose of 5 mg/kg using two different formulations—namely, free artemether solution formulated with cosolvency and artemether-loaded zein nanoparticles. The plasma artemether concentration–time data are shown in Figure 11, while the major intravenous pharmacokinetic parameters are listed in Table 2.

Similar to the results observed in a previous study [28], artemether was eliminated from plasma through a biexponential process—i.e., a rapid distribution phase followed by a prolonged terminal elimination phase. Therefore, the plasma artemether concentration–time data of the respective rat were fitted into the classical two-compartment first-order open model [29,30]. When it was given in free solution form, artemether was found to have a moderate apparent volume of distribution of the central compartment (*V_c_* = 2.14 ± 0.98 L/kg), a very rapid clearance (*CL* = 83.1 ± 17.4 mL/min/kg) and a moderate terminal elimination half-life (*t*_1/2 *λZ*_ = 167 ± 47 min) and mean residence time (*MRT* = 45.6 ± 16.4 min). When nanoparticles were given intravenously, although the *V_c_* value was similar (1.95 ± 0.85 vs. 2.14 ± 0.98 L/kg), artemether tended to display a longer *t*_1/2 *λZ*_, slower *CL* but more abundant plasma exposure. Of note, the nanoparticles led to ~80% increase in the *MRT* of artemether (82.9 ± 15.2 vs. 45.6 ± 13.4 min, two-tailed independent *t*-test: *p* = 0.0057). Moreover, at 300 and 420 min after administration, plasma artemether levels in rats that received nanoparticles were significantly higher than that in rats that received artemether solution (*p* < 0.05). Similarly, at 600 min after intravenous administration, artemether remained measurable in all rats received nanoparticles (13.8 ± 4.1 ng/mL); however, plasma level of artemether dropped to unmeasurable levels (<10 ng/mL) in all rats that received free artemether solution. Clearly, the extended-release effect of artemether-loaded zein nanoparticles was confirmed by in vivo study.

## 3. Discussion

According to the treatment guidelines issued by the WHO [7], parenteral artemisinin derivatives were used to manage severe malaria. Intravenous/intramuscular administration of water-soluble artesunate is the first choice. When parenteral artesunate is not available, an oil-based intramuscular injection of artemether can be used. Besides injection pain, the oil-based dosage form of artemether is associated with slow absorption and erratic pharmacokinetics, which adversely affect its clinical efficacy. In the present study, we formulated artemether into an intravenous dosage form using nanotechnology. For the management of life-threatening severe malaria, an intravenous formulation is obviously superior to an intramuscular dosage form as it leads to immediate onset antimalarial effects with higher and more consistent blood exposure, consequently decreasing the mortality. Our findings shed light on an innovative therapeutic option for severe malaria.

It is well known that artemisinin and its derivatives, including artesunate, artemether and dihydroartemisinin, are short-acting antimalarial agents that kill parasites more rapidly than other antimalarials, and are active against both the asexual and sexual stages of the parasite life-cycle [31]. The metabolism of artesunate and artemether have been well elucidated and both of them can be considered as a prodrug of dihydroartemisinin because they are rapidly hydrolyzed to dihydroartemisinin upon dosing [31]. It has been reported that artemether displayed a longer half-life than regardless of whether intravenous administration or oral dosing was used [28,32,33]. Since dihydroartemisinin, the active metabolite of artesunate/artemether, is also short-acting, a parent drug with a longer half-life is preferable as it will lead to a blood artemisinin derivative(s) level above the minimal effective concentration for a longer duration and reduced dosing frequency, consequently enhancing the therapeutic efficacy. From this angle, artemether appears to be a therapeutic entity superior to artesunate. However, in current clinical practice, intravenous artesunate, which commonly displays a half-life less than 1 h [33], is the first choice for severe malaria [7]. This is probably due to the solubility issue of artemether, which has hindered its intravenous administration. The artemether-loaded zein nanoparticles developed in the present study may enable intravenous delivery and change its role in the management of serve malaria.

The applications of nanoparticles to deliver artemether have been extensively attempted using different excipients and preparation methods and promising results were commonly reported [10,34,35,36,37,38,39,40,41,42]. However, most of the previous studies were focused on oral, transdermal or other nonparenteral delivery. So far, the intravenous dosage forms of artemether-loaded nanoparticles have only been attempted in a few studies using human serum albumin or glycerol trimyristate/monostearate plus soybean oil as carriers [10,39,40,43]. Again, such injectable formulations exhibited superior antimalarial efficacies and/or reduced toxicities in previous studies. However, as malaria is a disease of poverty [4,5], excipients with higher affordability such as zein and sodium caseinate are more cost-effective. To the best of the authors’ knowledge, this is the first attempt to use zein and sodium caseinate as major excipients to formulate artemether into a nanoformulation.

In the present study, the zein nanoparticles were found to release artemether through a biphasic process—i.e., a burst release phase followed by a slow-release phase. The burst release of artemether was probably due to the dissolution of unencapsulated artemether attached to the surface of the nanoparticles in PBS/bloodstream while the slow release was probably attributed to the release of artemether embedded in the core of the nanoparticles. As zein is hydrophobic, the penetration of water into the nanoparticles appears to be a slow process, resulting in the extended-release of artemether. As an intravenous dosage form, such biphasic release profile was favorable as it enabled immediate therapeutic effects upon administration and prolonged the therapeutic period.

Our explanations on the release profile were well supported by the pharmacokinetic data. When compared to the artemether delivered by cosolvent, artemether-loaded zein nanoparticles did not lead to an alternation in the *V_c_*. This phenomenon could be well explained by the burst release of artemether. As we knew, ~50% of artemether associated with the nanoparticles was not encapsulated, so upon intravenous administration, this portion of artemether would leave the nanoparticles and enter the bloodstream rapidly, resulting in a similar pharmacokinetic profile in the first hour after administration. In comparison to the artemether delivered by cosolvent, the formulation of nanoparticles substantially slowed down the decline of the plasma artemether level at terminal elimination stage and dramatically increased the *MRT*. The prolongation of residence of artemether could be explained by the extended-release of artemether from the nanoparticles, leading to a longer circulating period in bloodstream. An intravenous dosage form of artemisinin derivative with extended-release property is highly favorable as all these therapeutic agents suffer from rapid clearance and short half-lives, and prolonged infusion is required if the drug is given through an intravenous route. Hopefully, the artemether-loaded zein nanoparticles may decrease the dosing frequency and offer a more patient-friendly therapeutic option.

During the past two decades, nanomedicines have been extensively attempted in cancer therapy. Such approaches may increase drug accumulation through enhanced permeability and retention in tumors to improve anticancer efficacy and provide long systemic circulation of entrapped drug with high plasma concentration [44]. Similarly, our zein nanoparticles substantially prolonged the systemic circulation of artemether, an antimalarial agent with a very short half-life. However, the impact of zein nanoparticles on artemether accumulation in its target sites remains unclear. As artemisinin derivatives can kill the parasites at both human liver and blood stages [31], liver and red blood cells can be considered as their targets organ/cells. Although we attempted to measure artemether and its active metabolite dihydroartemisinin in red blood cells and plasma when we developed the assay, we only managed to quantify artemether in plasma. Therefore, whether artemether-loaded zein nanoparticles enhanced artemether accumulation remains unclear. Of note, in a previous study, human serum albumin nanoparticles substantially increased the penetration of artemether to RBCs and enhanced its antimalarial efficacy in mice [40]. Similarly, as zein nanoparticles enhanced liver uptake in various previous studies [24,45,46,47], it appears to be a practical strategy for hepatic targeting, which is highly favorable for the treatment of malaria.

In clinical management of malaria, hemolysis commonly occurred after artemisinin-based therapy [11]. A recent study suggested that the hemolysis was malaria-independent and mediated through a toxic oxidative effect of artemisinin derivative(s) on the red blood cell membrane as malaria-free rats receiving artesunate also suffered from hemolysis [48]. In the present study, we confirmed that free artemether was highly hemolytic in our in vitro test. Similarly, the excipient sodium caseinate also caused hemolysis. Interestingly, when artemether and sodium caseinate were formulated into zein nanoparticles, their hemolytic effects were substantially masked. Probably through the formation of nanoparticles, sodium caseinate and ~ half of the artemether were sealed inside the nanoparticles, minimizing the amounts of free sodium caseinate and artemether. Moreover, in our pharmacokinetic study, hemolysis was not observed in blood samples collected from rats receiving artemether-loaded zein nanoparticles but observed in some rats receiving artemether solubilized in cosolvent (data not shown). Clearly, artemether-loaded zein nanoparticles were less hemolytic. However, the beneficial effect of artemether-loaded zein nanoparticles on hemolysis needs to be confirmed in a study where the animals receive repeated dosing as artemisinin associated hemolysis commonly had a delayed onset after repeated dosing in clinical settings [11].

Due to the constrained resources, the antimalarial effects of artemether-loaded zein nanoparticles were not attempted in the present study. It would be of great scientific interest to assess its parasite-killing activities in cell culture models and examine therapeutic efficacy in malaria-bearing rodents. Similarly, the preclinical safety/toxicological profile should be carefully examined.

In summary, an innovative intravenous dosage form of artemether was formulated using artemether-loaded zein nanoparticles. This formulation was found to display good encapsulation efficiency, excellent physical stability and offer extended-release property. Encapsulation of artemether into zein nanoparticles strongly suppressed in vitro hemolysis. Upon intravenous administration, artemether-loaded zein nanoparticles substantially prolonged the circulation of artemether in the bloodstream, suggesting that the nanoparticles may enhance its therapeutic efficacy in clinical settings. In conclusion, intravenous delivery of artemether-loaded zein nanoparticles appears to be a promising therapeutic option for the management of severe malaria.

## 4. Materials and Methods

### 4.1. Materials

Artemether was supplied by Biotain Pharma Co., Ltd. (Xiamen, China). Zein was a kind gift from Flo Chemical Corporation (Ashburnham, MA, USA). Sodium caseinate was purchased from Sigma-Aldrich (St. Louis, MO, USA). Absolute ethanol was obtained from VWR Singapore Ltd. (Singapore). Ultrapure water was prepared by a Elix^®^ Essential 5 UV Water Purification System (Molsheim, France) or a Millipore Direct-Q^®^ Ultra-Pure Water System (Billerica, MA, USA) and used in all experiments. High performance liquid chromatography (HPLC) grade acetonitrile was supplied by Tedia (Fairfield, OH, USA). All other chemicals are of reagent grade and were obtained from either Sigma-Aldrich or Tokyo Chemical Industry (Tokyo, Japan).

### 4.2. Preparation of Artemether-Loaded Zein Nanoparticles

Artemether-loaded zein nanoparticles were prepared by a modified antisolvent precipitation approach [14]. Briefly, zein (50–300 mg) and artemether (0–30 mg) were dissolved in 5 mL of the ethanol–water binary solvent system containing 50, 60, 70, 80, and 90% *v*/*v* ethanol. Sodium caseinate (0–150 mg) was dissolved in 10 mL water. In total, 10 mL of aqueous sodium caseinate solution was added instantly to 5 mL zein solution with a 1000 rpm magnetic stirring. Upon mixing, artemether-loaded zein nanoparticles precipitated immediately. Ethanol was then removed from the nanosuspension either by normal evaporation with magnetic stirring or rotary evaporation (Rotavapor^®^ R-205, BUCHI Labortechnik AG, Flawil, Switzerland) for 1–5 h. The prepared nanosuspension was frozen in liquid nitrogen and lyophilized for 48 h. The achieved artemether-loaded zein nanoparticles powders were stored at 4 °C until further analysis.

### 4.3. Particle Size and Zeta Potential Analyses

Particle size of artemether-loaded zein nanoparticles was analyzed by dynamic light scattering (DLS) using Zetasizer Nano ZS (Malvern, UK) [10]. In brief, nanoparticles were dispersed in water to make the equivalent of 0.2 mg zein/mL. Scattering angle and temperature were set to 175° and 25 °C, respectively. Each sample was equilibrated for 120 s and data were collected over 5 sequential readings. Measurements were performed in triplicate. Mean particle size (Z-average, diameter) and standard deviation were calculated. Zeta potential was measured by electrophoretic light scattering technology using Zetasizer Nano ZS (Malvern, UK). Nanoparticles were diluted to the equivalent of 0.2 mg zein/mL, as was carried out for particle size analysis. Measurements were carried out in triplicate and the mean and standard deviation were calculated.

### 4.4. Drug Content and Encapsulation Efficiency

To obtain the total artemether content in the lyophilized artemether-loaded zein nanoparticles powder, 90% *v*/*v* ethanol–water binary solvent system was used to reconstitute the powder. After sonication for 30 min, the suspension was centrifuged at 10,800 rpm for 30 min. The supernatant was collected and assayed using high performance liquid chromatography (HPLC) analysis. A Zorbax Eclipse C18 reversed phase column (4.6 × 150 mm, 5 μm, Agilent, USA) was used with 70% acetonitrile plus 30% 0.01 M KH_2_PO_4_ solution (pH 4) as the mobile phase. Drug content was calculated using the formula below:

(1) $$Drug\;Content\;\left(\%\right)=\frac{Amount\;of\;artemether\;in\;nanoparticles}{The\;amount\;of\;nanoparticles}\times 100\%$$

To evaluate the encapsulation efficiency, ethyl acetate was used to extract unencapsulated artemether from the artemether-loaded zein nanoparticles powder [10]. Ethyl acetate was the solvent of choice since artemether is soluble in it but zein is not [14]. Accurately weighed artemether-loaded zein nanoparticle powder was dispersed in 1 mL ethyl acetate for 1 min. The supernatant was collected and analyzed in the same way as the drug content. The amount of artemether encapsulated was calculated by deducting the amount of unencapsulated artemether from the drug content in the artemether-loaded zein nanoparticles. Encapsulation efficiency (EE) was calculated using the formula below:

(2) $$Encapsulation\;efficiency\;\left(\%\right)=\frac{Amount\;of\;encapsulated\;artemether}{Total\;amount\;of\;artemether\;in\;zein\;nanoparticles}\times 100\%$$

### 4.5. Particle Morphology

The morphology of artemether-loaded zein nanoparticles was observed using field emission scanning electron microscope (FESEM, JEOL JSM-6700F, Tokyo, Japan) at a 5 kV acceleration voltage [10]. Prior to visualization, the sample was placed on double sided copper tape mounted on metal stubs. It was then sputtered with gold using Cressington 208HR (Ted Pella, Inc., UK) at 10 mA for 120 s.

### 4.6. Thermal Analysis

Differential scanning calorimetry (DSC) was employed to study the thermal properties of artemether-loaded zein nanoparticles powder, raw artemether, zein, sodium caseinate and their physical mixture at the mass ratio 1:5:5 [10]. Diffractograms were taken using a Mettler Toledo DSC 1 (Mettler-Toledo AG, Analytical, Schwerzenbach, Switzerland). Samples of 2–5 mg were sealed in an aluminium crucible and heated at a rate of 10 °C/min over a temperature range of 30 to 120 °C under 10 mL/min N_2_.

### 4.7. Crystallinity

Crystallinities of artemether-loaded zein nanoparticles powder, raw artemether, zein, sodium caseinate and their physical mixture at mass ratio 1:5:5 were investigated using a powder X-ray diffractometer (D8-Advance, Bruker AXS GmbH, Karlsruhe, Germany) with a PSD Vantec-1 detector [10]. Data were acquired over an angular range of 2°–40° (2θ) at a step of 0.017° using monochromatized CuKα radiation (λ: 1.542 Å) with 20 kV and 40 mA. Divergence and antiscattering slits were set at 0.3°.

### 4.8. Drug–Protein Interaction

Fourier transform infrared (FTIR) spectra of artemether-loaded zein nanoparticles powder, raw artemether, zein, sodium caseinate and their physical mixture at a mass ratio of 1:5:5 were acquired using an Excalibur FTS 3000 MX (Bio-Rad, Hercules, CA, USA) [10]. The sample to be investigated was mixed with KBr at a mass ratio 1:100 and compressed to form a disc prior to FTIR scanning. Pure KBr disc was used as the background. For each measurement, 64 scans were taken at a spectral resolution of 4 cm^−1^, over a wavenumber range of 400 to 4000 cm^−1^.

### 4.9. In vitro Release Study

The dissolution profile of artemether-loaded zein nanoparticles was studied by the “sample and separate” method [49]. In brief, artemether-loaded zein nanoparticle powders containing 6 mg artemether were put in 100 mL phosphate buffered saline (PBS) of pH 7.4. The study was performed under sink conditions at 37 ± 0.5 °C with 150 rpm stirring. The beaker was covered with parafilm to minimize water loss via evaporation. In total, 1 mL samples were withdrawn at selected time points (1/12, 1/6, 1/4, 1/2, 1, 2, 3, 4, 5, 6, 24 h). Samples were centrifuged at 10,800 rpm for 30 min and the supernatants were analyzed by HPLC in the same way as drug content. Then, 1 mL PBS at 37 °C was added after each sampling to maintain sink condition. The experiment was repeated 3 times to ensure consistency. Raw artemether was used as control. Mean values and standard deviations were subsequently calculated.

### 4.10. In Vitro Hemolytic Test

This test was performed to examine the hemolytic potential of artemether-loaded zein nanoparticles and the formulation ingredients on red blood cells (RBCs) [10]. Freshly collected rat blood was centrifuged at 1500× *g* for 10 min at 4 °C. The plasma was collected for other purpose. The remaining RBCs were washed with PBS and then centrifuged at 1500× *g* at 4 °C for 5 min. After two repeats of such procedures, the RBCs were diluted 25 times with PBS to make a 4% *v*/*v* RBCs dispersion. Such RBCs were kept at 4 °C and used within 24 h.

The impact of artemether-loaded zein nanoparticles containing 2 mg/mL artemether, raw artemether in 5% *v*/*v* aqueous DMSO solution (2 mg/mL), 5% *v*/*v* aqueous DMSO solution, sodium caseinate (7.5 mg/mL) solution, artemether (2 mg/mL) together with sodium caseinate (7.5 mg/mL) solution was assessed.

PBS and 1% *v*/*v* Triton-X 100 solution were assessed by adding 1 mL of the test solutions each to 1 mL of the 4% *v*/*v* RBCs dispersion. Each sample was incubated with the RBCs at 37 °C for 1 h and then centrifuged at 1500× *g* for 10 min. The absorbance of the supernatant was taken at 550 nm with U-2900 UV spectrophotometer (Hitachi, Japan). Percentage haemolysis was calculated as follows:

(3) $$Hemolysis\;\left(\%\right)=\frac{Absorbance_{Test\;group}-Absorbance_{PBS}}{Absorbance_{Triton-X100}-Absorbance_{PBS}}\times 100\%$$

### 4.11. Physical Stability

Artemether-loaded zein nanoparticles were stored at 4 °C and 75% relative humidity and monitored for changes in particle size, zeta potential and encapsulation efficiency for 24 weeks. Samples were taken out at predetermined time points for particle size, zeta potential and encapsulation efficiency were examined. Stability of artemether-loaded zein nanoparticles after reconstitution with Milli-Q water or normal saline upon storage at 4 °C was studied by measuring particle size and zeta potential. Samples were taken at predetermined time points for particle size and zeta potential analyses over a 7 h period.

### 4.12. Pharmacokinetic Study

This pharmacokinetic study was conducted with strict adherence to the Guidelines on the Care and Use of Animals for Scientific Purposes (Singapore). The study design and animal handling procedures were reviewed and approved by the Institutional Animal Care and Use Committee of the National University of Singapore (NUS) (Project No.: R15–1273, 5 November 2015). All in vivo experiments were carried out in a specific pathogen-free animal facility (temperature: 22 ± 1 °C; humidity: 60–70%) in Comparative Medicine, NUS [50,51]. Male Sprague–Dawley rats (9–10 weeks old, weight: 300–350 g) were ordered from InVivos (Singapore) through Comparative Medicine. The animals were housed under a 12 h light–dark cycle with free access to food and water. On the day before the pharmacokinetic study, surgery was performed and a catheter (polyethylene tube, i.d. = 0.580 mm, o.d. = 0.965 mm, Becton Dickinson, Sparks, MD, USA) was implanted into the right jugular vein under isoflurane anesthesia. Intravenous artemether administration and blood collection were carried out via this cannula. To prevent cross-contamination and blood clotting, ~0.3 mL heparin-saline (10 I.U./mL) was flushed through the cannula after intravenous artemether administration or each blood sampling. This reliable model has been routinely used in our laboratory to assess preclinical pharmacokinetics [52,53,54].

The intravenous pharmacokinetic profiles of artemether were subsequently examined in rats using two different formulations—namely, free artemether solution formulated with cosolvency and artemether-loaded zein nanoparticles. Artemether solution (5 mg/mL) was prepared in Cremophore EL-saline (1:3) as reported in a previous pharmacokinetic study [28], while the dry powder of nanoparticles was reconstituted in isotonic saline to a final artemether concentration of 5 mg/mL. Ten rats were divided into two groups. Group 1 (*n* = 5) received a single bolus intravenous injection of free artemether solution at 5 mg/kg, while Group 2 (*n* = 5) received the same dose of artemether in artemether-loaded zein nanoparticles. Serial blood samples were collected from both groups before dosing and at 5, 15, 30, 60, 90, 120, 180, 300, 420, 600 and 1440 min after intravenous injection. To enhance the stability of artemether, 1% *v*/*v* of 0.4 M potassium dichromate was added to heparinized tubes that would be used to collect rat blood samples [55]. After centrifugation at 5000× *g* for 5 min, the plasma samples were harvested and stored at −40 °C until liquid chromatography–tandem mass spectrometry (LC–MS/MS) analysis.

### 4.13. LC–MS/MS Analysis

The LC–MS/MS system, consisting of an Agilent 1290 Infinity Liquid Chromatography system (Agilent Technologies, Santa Clara, CA, USA) and an ABSciex QTRAP 5500 mass spectroscopy (AB Sciex, Framingham, MA, USA) equipped with TurboIon Spray probe (AB Sciex), was applied to quantify the plasma level of artemether. Analyst 1.6.2 software (AB Sciex) was used for the operation of the LC–MS/MS system and for data analyses. Nitrogen was used as nebulizing, curtain and collision gases. The ion source was operated in the positive mode. Mass spectrometer parameters including curtain gas, gas 1, gas 2 pressures were set at 20, 40 and 40 psi, respectively. Temperature and ion spray voltage were set at 600 °C and 5500 V, respectively. The precursor-to-product ion transition for artemether was *m*/*z* 316 → 267 and for artemisinin (internal standard) this was *m*/*z* 300 → 135 [56]. Optimal compound parameters, namely the declustering potential, entrance potential, collision energy and collision exit cell potential, obtained were 46.00, 10.00, 12.00 and 22.00, respectively, for *m*/*z* 316 → 267 and 85.00, 12.00, 34.58 and 24.00, respectively, for *m*/*z* 300 → 135.

Chromatographic separations were performed using a reversed-phase column (Agilent Poroshell 120 EC-C18: 75 × 3.0 mm, 2.7 μm) with guard column (Agilent Poroshell 120 EC-C18: 5 × 3.0 mm, 2.7 μm). Acetonitrile was used as the organic phase and aqueous solution containing 0.04 M ammonium acetate and 0.1% *v*/*v* formic acid was used as the aqueous phase. Gradient delivery of the mobile phase at a flow rate of 0.35 mL/min at room temperature (~24 °C) was used for the chromatographic separation. The gradient schedule used was (i) 0.00–0.50 min, acetonitrile 50%; (ii) 0.50–6.50 min, acetonitrile 50 → 95%; (iii) 6.50–6.60 min, acetonitrile 95 → 50%; (iv) 6.60–8.00 min, acetonitrile 50%. MS was operated in multiple reaction monitoring (MRM) mode at a unit mass resolution with a dwell time of 100 ms. The calibration curve ranged from 10 to 1000 ng/mL artemether (0.017–3.356 nM).

A protein precipitation procedure was employed to clean plasma and extract artemether [57,58]. Internal standard working solution was prepared by diluting stock solution of artemisinin with acetonitrile and then spiking with formic acid to obtain 1000 ng/mL artemisinin solution with 1% *v*/*v* formic acid. During sample preparation, three volumes of internal standard working solution were then added to one volume of rat plasma, vortexed, and centrifuged at 10,000× *g* for 10 min at 4 °C. The supernatant was put into a 250 μL plastic autosampler vial (Agilent Technologies) for analysis. For each assay, 10 μL of sample was injected into the LC–MS/MS system.

### 4.14. Pharmacokinetic Calculation

All pharmacokinetic analyses were carried out using WinNonlin standard version 1.0 (Scientific Consulting Inc., Apex, NC, USA). Since the log plasma artemether concentration–time curves of all rats receiving intravenous administration displayed biexponential decline, a classical two-compartment first-order open model was used to represent the intravenous pharmacokinetic profile and the apparent volume of distribution of the central compartment (*V_c_*) was subsequently calculated [29,30]. The plasma exposure (area under the plasma concentration–time curve from 0 min to last measurable point (AUC)) was calculated by the log trapezoidal method. Clearance (*CL*), mean residence time (*MRT*) and terminal elimination half-life (*t*_1/2 *λZ*_) were also calculated with a noncompartmental method [29,30]. A weighting factor of 1/*y*^2^ was adopted in all pharmacokinetic modeling [59].

### 4.15. Statistics

All experiments were performed in triplicate unless otherwise stated. The results are expressed as mean ± standard deviation (SD). The difference between means of two groups were analyzed by two-tailed unpaired *t*-test. Statistically significant differences were indicated by *p* values less than 0.05.

## Figures and Tables

**Table 1:** Artemether content and Encapsulation Efficiency (EE) *.

Zein/Artemether Ratio	Artemether Content (% *w*/*w*)	Encapsulated Drug (% *w*/*w*)	EE (%)
2.5:1	16.3 ± 0.7	5.07 ± 0.32	31.0 ± 2.4
3.3:1	12.8 ± 0.4	5.86 ± 0.31	45.9 ± 1.9
5:1	8.90 ± 0.20	4.64 ± 0.17	52.1 ± 2.0
10:1	4.60 ± 0.20	2.53 ± 0.17	56.6 ± 2.5
20:1	2.42 ± 0.09	1.51 ± 0.07	62.3 ± 3.0

* EE is the ratio between the amount of encapsulated artemether and the total amount of artemether in nanoparticles; Data are presented as mean ± SD (*n* = 3).

**Table 2:** Intravenous pharmacokinetic parameters of artemether ^a^.

Formulation	Artemether Solubilized in Cosolvent	Artemether-Loaded Zein Nanoparticles
Dose (mg/kg)	5	5
*V_c_* (L/kg)	2.14 ± 0.98	1.95 ± 0.85
*AUC*_0→*last*_ (10^4^× min·ng/mL)	6.22 ± 1.22	8.14 ± 4.52
*CL* (mL/min/kg)	83.1 ± 17.4	72.4 ± 25.7
*t*_1/2 *λZ*_ (min)	165 ± 47	297 ± 132
*MRT*_0→*last*_ (min)	45.6 ± 16.4	82.9 ± 15.2 **

^a^ Results are presented as Mean ± SD (*n* = 5); ** *p* < 0.01 between artemether-loaded zein nanoparticles and artemether solubilized in cosolvent.

## Data Availability

The data presented in this study are available on request from the corresponding authors.

## Abbreviations

*CL*	Clearance
DMSO	Dimethyl sulfoxide
DSC	Differential scanning calorimetry
FTIR	Fourier transform infrared
HPLC	High performance liquid chromatography
LC–MS/MS	Liquid chromatography-tandem mass spectrometry
*MRT*	Mean residence time
nm	nanometer
PBS	Phosphate buffer solution
PDI	Polydispersity index
pI	Isoelectric point
RBCs	Red blood cells
SD	Standard deviation
*t*_1/2 *λZ*_	Terminal elimination half-life
*V_c_*	Apparent volume of distribution of the central compartment
WHO	World Health Organization
